# Effect of Light-Emitting Grid Panel on Indoor Aquaculture for Measuring Fish Growth †

**DOI:** 10.3390/s24030852

**Published:** 2024-01-28

**Authors:** Nguyen Ngoc Huynh, Myoungjae Jun, Hieyong Jeong

**Affiliations:** Department of Artificial Intelligence Convergence, Chonnam National University, Gwangju 61186, Republic of Korea; ngocnh2226@jnu.ac.kr

**Keywords:** segmentation, deep learning, fish growth, size, and length estimation, aquaculture

## Abstract

This study is related to Smart Aqua Farm, which combines artificial intelligence (AI) and Internet of things (IoT) technology. This study aimed to monitor fish growth in indoor aquaculture while automatically measuring the average size and area in real time. Automatic fish size measurement technology is one of the essential elements for unmanned aquaculture. Under the condition of labor shortage, operators have much fatigue because they use a primitive method that samples the size and weight of fish just before fish shipment and measures them directly by humans. When this kind of process is automated, the operator’s fatigue can be significantly reduced. Above all, after measuring the fish growth, predicting the final fish shipment date is possible by estimating how much feed and time are required until the fish becomes the desired size. In this study, a video camera and a developed light-emitting grid panel were installed in indoor aquaculture to acquire images of fish, and the size measurement of a mock-up fish was implemented using the proposed method.

## 1. Introduction

Koreans’ annual consumption of marine products per person is 68 kg, the world’s No. 1 country (as of 2018, OECD 2020 report) (the report is at https://www.oecd.org/country/korea, accessed on 14 November 2023). Moreover, since Korea is highly dependent on the fishing industry, developing fish food resources is essential in securing Korea’s food resources in the future. However, as Korean society is entering an ultra-aged society, the number of fishermen is rapidly aging, the shortage of human resources at the fisheries site is intensifying, and the environment of the fisheries sector is becoming a workforce that young people avoid. In addition, amid growing concerns that changes in the fishing environment and unexpected disasters caused by climate change in each country could cause food shortages, management is worsening due to a decrease in fishing production and a high-cost and low-efficiency fisheries structure [1,2,3].

Since fish are food resources that can produce the highest amount of protein per unit area, developing technologies related to fish farming can be an essential cornerstone for securing future food resources. The general process of aquaculture is that fish (young fish that have just broken from eggs) are purchased, raised in the fish farm for a certain period, and then shipped when they are large enough to be commercially available. For example, a halibut fish (flatfish) farm usually operates dozens of 5 m round tanks. About 1000 halibut are farmed in one tank, and farming managers regularly sample and measure the size of the fish, roughly determine the feed supply, and decide the feed supply plan and ship it. In order to automate the process of these fish farms, a technology that can automatically measure the size of fish is first required. The size of fish is an important indicator that determines the marketability of fish and an important indicator that determines the amount of feed needed for farming, so it is an essential technology for automating the entire farming process [4,5,6].

By using cameras to capture high-quality fish information, machine vision can be applied to aquaculture to lessen the workload for fishermen. This methodology is referred to as the digital imaging method. Take information using the camera and use the expected features. The expectation of lengths was previously determined by researchers mostly from fish data; few employed cameras for this purpose. Thus, to increase the effectiveness of the picture length measurement procedure, we must digitize the images and create image processing software [7,8].

Initially, the measurement of fish growth is performed manually. Namely, it is necessary to bring the fish out of the water to measure the fish size. Because it is only possible to measure some fish in indoor aquaculture, only a few samples have been measured for the estimation. Recently, image processing technology that combines artificial intelligence (AI) technology has developed significantly [9,10,11], so technology measuring the size of living things by camera images is possible [12,13,14]. However, in the current indoor aquaculture farm, measuring the size only by acquiring images with the only camera is not easy [15,16,17]. The main reason is related to the lighting condition. The domestic fish farm uses only natural lighting when operators feed them without special lighting. Thus, it is dark, and the fish in the aquaculture farm overlap, which is difficult to identify only by camera images [18,19].

Therefore, this study aims to develop a light-emitting grid panel to apply for an indoor aquaculture farm to overcome the light conditions. Then, it verifies that the developed grid is helpful for the automatic measurement of size and area for the fish with a high accuracy of over 90%. Experimental results showed that light-emitting grid panels helped to measure fish growth even in dark environments. This study’s results can be fundamental in providing a biological growth measurement system for innovative modalities that predict feed supply amount and time. Previously, the OCEANS Conference focused on hardware description, and this study has focused on software development. Specifically, the following parts were added to the software: determination of whether the fish is within the grid, judgment of whether the fish are overlapping or only one fish is separated, detection of a single fish, segmentation of the found fish, and measurement of the pixel area, measurement of the pixel length through the bounding box, and finally unit conversion.

Our main contributions are as follows:

- We proposed the light-emitting grid panel as the metric to estimate the size of an object in an image in any condition, including low lighting, without collecting any additional information.
- The real-time measurement of the fish size and count enabled us to evaluate the fish growth and recognize the feeding amount and living environment.

## 2. Materials and Methods

### 2.1. Experimental System

**Figure 1:**
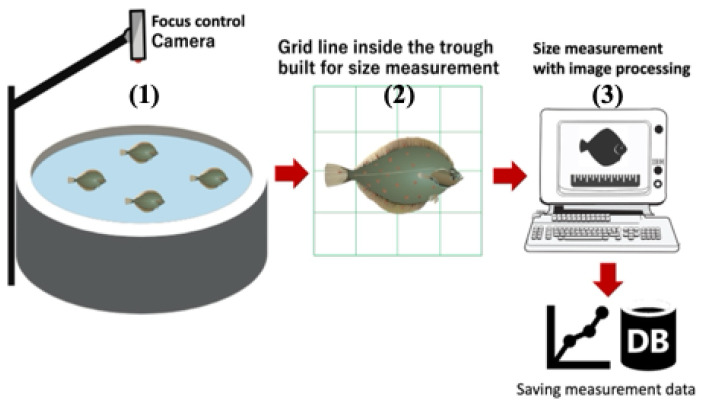
An overall system concept and role of the proposed artificial intelligence of things (AIoT) device toward the automatic unmanned management in the indoor aquaculture farm: the single camera (1) is installed near the ceiling to capture the whole range, the light-emitting grid panel (2) is set to the bottom of the water tank, and it is possible to decide on the shipment after the prediction of the feed amount and time is analyzed automatically with acquired data (3).

Figure 1 shows the overall system concept and the proposed artificial intelligence of things (AIoT) device’s role in automatic unmanned management in the indoor aquaculture farm. The single camera (1) is installed near the ceiling to capture the whole range, and the light-emitting grid panel (2) is set to the bottom of the water tank, and it is possible to decide on the shipment after the prediction of the feed amount and time is analyzed automatically with acquired data (3).

(1): There is an imaging camera installed at the top of the water tank. The image data acquired by the camera are transmitted to the computer through the Ethernet cable. The central computer can manipulate the camera’s movement and analyze the video data obtained from the camera to estimate the length of the fish. In addition, the central computer periodically uploads the analyzed data to the web server to monitor the growth status of the fish in the web browser state. The image output from the camera is 4K resolution and is transmitted to the computer at 60 frames per second (fps) at a size of 3840 × 2160. The camera’s pen, tilt, and optical zoom functions focus the camera on the grid panel (2) and zoom in until the grid panel (2) is complete on the monitor screen. The reason is to obtain the highest quality image source, if possible. The computer (3) saves the video for 30 s as the fish passes through the grid panel.

(2): The light-emitting grid panel is installed on the bottom surface of the reservoir. When a fish passes over the grid, a camera installed in the fish tank transmits a fish image to the computer (3), and the computer (3) estimates the length by distinguishing the shape of the fish through a fish length estimation algorithm. The relationship between the actual length of the grid panel and the pixels is known in advance; thus, pixel-size data can be converted into the millimeter (mm) unit [14,20]. The actual length of the manufactured grid panel is 10 mm. In addition, since the segmented area of the creature in the grid panel consists of pixels, the area and length can be estimated through unit conversion. The relationship with weight per length can be derived when multiple samples are collected.

(3): The computer stores the images and calculates the size, area, and number of fish within the grid area in real time. First, fish that enter the grid are found in the video, and segmentation is performed using a model trained by the author in advance. In the segmentation results of the fish area, the total number of pixels represents the fish area. Thus, this information is displayed above the bounding box to inform the user. Next, the location and orientation of the fish are determined, and the bounding box is adjusted according to the location and orientation of the fish. Through the process, the long side of the bounding box represents the fish’s size, and the short side represents the fish’s width.

### 2.2. Development of Light-Emitting Grid Panel

**Figure 2:**
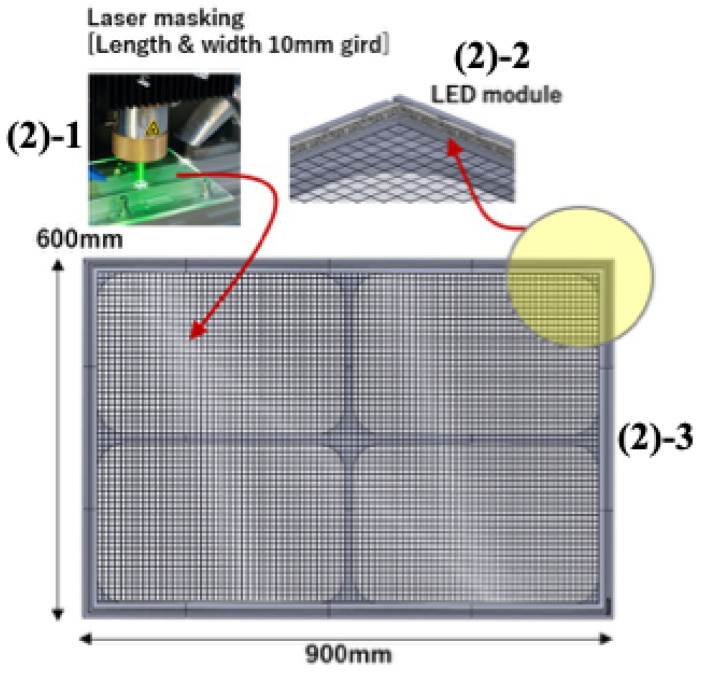
A proposed light-emitting grid panel to acquire the fish image for measuring the growth in the dark environment of aquaculture and then convert the pixel unit to the millimeter unit from the captured fish image.

Figure 2 is a proposed light-emitting grid panel to acquire the fish image for measuring the growth in the dark environment of aquaculture and then convert the pixel unit to the millimeter unit from the captured fish image. The manufactured panel is (width × height = 900 × 600 mm). The panel size can be changed according to the installation environment.

(2)-1: To release grid lines, a 10T thick transparent acrylic plate is laser-masked with grid patterns in 10 mm width and height. When the LED light is irradiated from the side of the masked acrylic plate, the light is reflected along the grid line, creating the same effect as the grid line emitting light throughout the acrylic plate. The generated grid line is the same principle that a laser light source emits light by creating a total reflection along the optical fiber. The total reflection effect refers to the phenomenon in which the light generated by the laser light source is reflected on the object and dispersed throughout. Unlike ordinary light sources, lasers proceed in a straight line and have intense concentration, characterized by a straight line of light and the concentration of light from the light source in a particular direction. However, as it hits the object, it is reflected at a certain angle and distributed in various directions, resulting in a total reflection effect of uniformly distributing the brightness.

(2)-2: The light source used in this study is a general LED light source, not a laser light source. However, to create a total reflection effect, LED light is irradiated from the grid panel’s top, bottom, left, and right directions. The LED light emitted from the four directions reflects along the grid lines distributed throughout the grid panel. It emits light, inducing a phenomenon similar to the total reflection effect when the laser light source passes through the optical fiber. The light-emitting grid panel is switched on with the camera only at certain times to acquire image information and off at other times. The used LEDs are a combination of UV color LEDs and UV LEDs. Color LEDs are used because the frequency band of the favorite color varies depending on the type of fish. Furthermore, UV LEDs are used because ordinary fish have the characteristic of liking UV light sources.

(2)-3: The frame supporting the LED grid panel is made using 3D printing and assembled into four parts. Inside the frame, there is a space for securing the LED module, and the LED module uses 12 V DC power. The LED panel’s brightness and power on/off are controlled in the external control mode.

## 3. Real-Time Measurement

### 3.1. Experimental Environment

**Figure 3:**
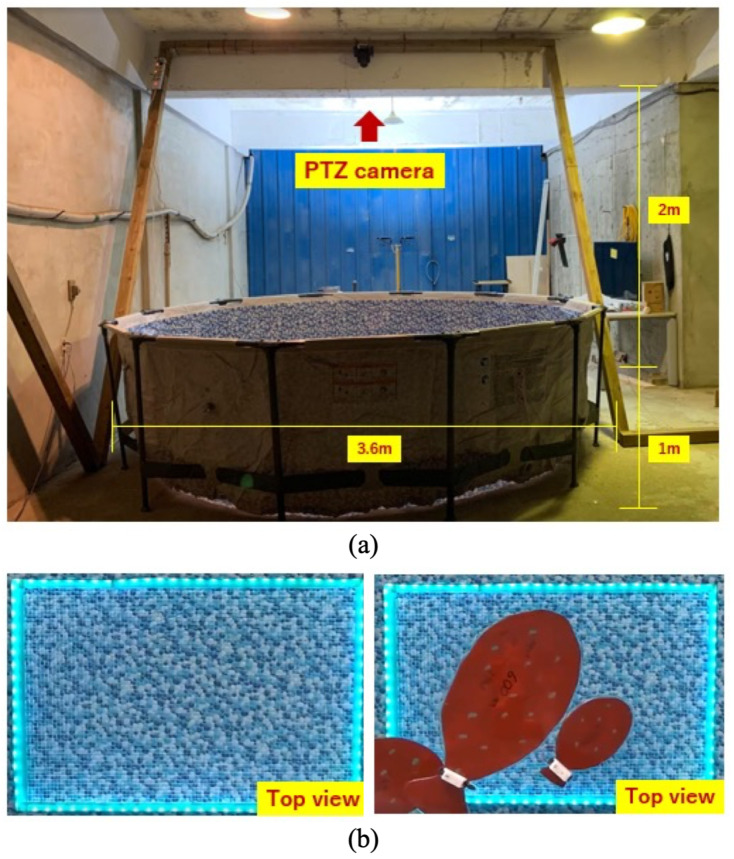
An overview of the experimental environment consisting of a circular water tank, a PTZ (pan-tilt-zoom) camera on the ceiling, and the developed grid panel on the bottom of the tank (**a**), and the results of the acquired image with or without the mockup fish in the grid panel using the PTZ camera (**b**).

Figure 3 shows an overview of the experimental environment consisting of a circular water tank, a PTZ (pan-tilt-zoom) camera on the ceiling, and the developed grid panel on the bottom of the tank (a), and the results of the acquired image with or without the mockup fish in the grid panel using the PTZ camera (b). When workers evaluate the fish’s growth, measuring the real fish with the tapeline after taking the fish out of the water tank is necessary. Although fishes like the dark environment, the light turns on because humans must work in a bright environment. The critical point of the proposed experimental system is that the grid panel can evaluate the fish growth in a dark environment in real time.

The experiment is conducted in a circular water tank with a diameter of 3.6 m and a depth of 1 m. A PTZ (pan-tilt-zoom) camera is installed 2 m above the top and center of the tank for the experiment, and a mockup fish is manufactured for the experiment. Five different-size fish models are prepared; the actual sizes are 600, 450, 448, 315, and 270 mm, respectively. The movement of the fish is replaced by circulating the water using a water pump in the tank. The fish models rotate in the direction of water rotation and pass over the installed light-emitting grid panels at the bottom of the water tank. The video of the fish taken with the camera is transferred to the computer, and the size is measured through image segmentation. The experiment uses five fish models at 60 fps (frame per second) every 30 s. The AI tool used YOLOv8, one of the deep learning models. YOLO families are designed to reduce the model size and perform well on the CPU by applying lightweight technology; thus, it can be used to lighten systems using single-board computers such as Raspberry Pi. In this experiment, we rely entirely on YOLOv8 [21] to segment objects before estimating growth based on images. To test the effectiveness of the light-emitting panel as a metric in the image to estimate fish growth, we fine-tune the model to match our data.The point we focus on in this study is to develop the panel and verify the effect before applying the developed panel to the actual farm to save cash, time, and labor.

### 3.2. Method

**Figure 4:**
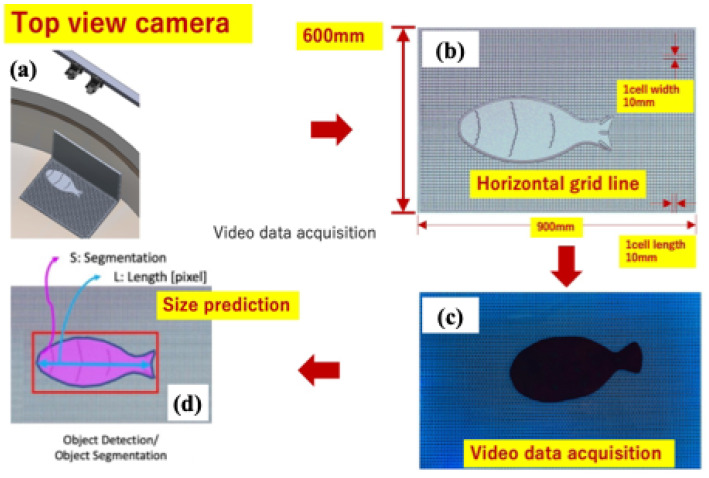
A process for measuring the fish growth in the dark environment: it is necessary to capture the fish image on the grid panel at first (**a**). The area of the fish image is calculated by counting the number of pixels (**b**), then the area can be converted from the pixel unit to the millimeter’s square unit based on the relationship between the pixel and the millimeter unit for the grid panel (**c**). Finally, the longest length of the bounding box can be equal to the length of the fish size (**d**).

Figure 4 shows the process for measuring fish growth in a dark environment. (a) indicates the experimental system (1) and (b) indicates the experimental system (2) in Figure 1. It is necessary to first capture the fish image on the grid panel at (c). (d): The area of the fish image is calculated by counting the number of pixels. Then, the area can be converted from the pixel unit to the millimeter’s square unit based on the relationship between the pixel and the millimeter unit for the grid panel. Finally, the bounding box’s most extended length can equal the fish size’s length. Images of the fish taken by the camera have the (width × height = 900 × 600 mm) light-emitting grid panel (a one grid length: width × height = 10 × 10 mm) background. The background screen contrasts the dark-colored fish because it emits light. The color of the grid panel can be adjusted to various colors, but in this study, a blue LED light source with good temporary facilities is used in the dark. The captured image is delivered to the computer in real time via Ethernet communication. The central computer records images every specific cycle and for 30 s in the MP4 format in the computer. After we complete the segment step, we can obtain the objects’ masks. Since we know the actual size (one space—width: 10 mm, length: 10 mm) of a square on the grid, it is possible to convert from the obtained pixel image of the fish to the actual size. The conversion is performed through Equations (1) and (2). In addition, after performing the estimation of the fish, we also develop a count of the number of fish inside the grid based on the coordinates of the objects that have just been segmented. By this method, we can check if the fish and grid overlap (IoU) with each other based on the detected coordinates and the coordinates of the original grid.

(1) $$\mathrm{Pixels}\;\mathrm{per}\;\mathrm{metric}=\frac{\mathrm{Size}\;\mathrm{of}\;\mathrm{grid}\;\mathrm{in}\;\mathrm{real}}{\mathrm{Size}\;\mathrm{of}\;\mathrm{grid}\;\mathrm{in}\;\mathrm{pixels}}$$

(2) Estimatedsize=(Pixelspermetric)×(Pixelsofobjects)

We need to assess their detection accuracy to determine how well our processing pipeline has generated the masks. The evaluation metrics commonly used in object detection, which include intersection over union (IoU) and pixel accuracy, are used to measure the segmentation results. The IoU, or the Jaccard index, is an evaluation metric that measures how accurately an object has been segmented in a given dataset. Typically, the IoU is calculated by comparing the bounding box predicted by the CNN detector with the ground truth bounding box manually labeled. In our case, the detector generates a pixel region (i.e., mask) that contains the pixels corresponding to a fish, and the ground truth is also a hand-labeled pixel region. As a result, we compute IoU by comparing these two pixel regions. To obtain the final score, we divide the area of overlap between the predicted and ground-truth regions by the area of the union of both regions.

(3) $$\mathrm{IoU}=\frac{\mathrm{ground}-\mathrm{truth}\cap \mathrm{prediction}}{\mathrm{ground}-\mathrm{truth}\cup \mathrm{prediction}}$$

## 4. Results

### 4.1. Results of Instance Segmentation

**Figure 5:**
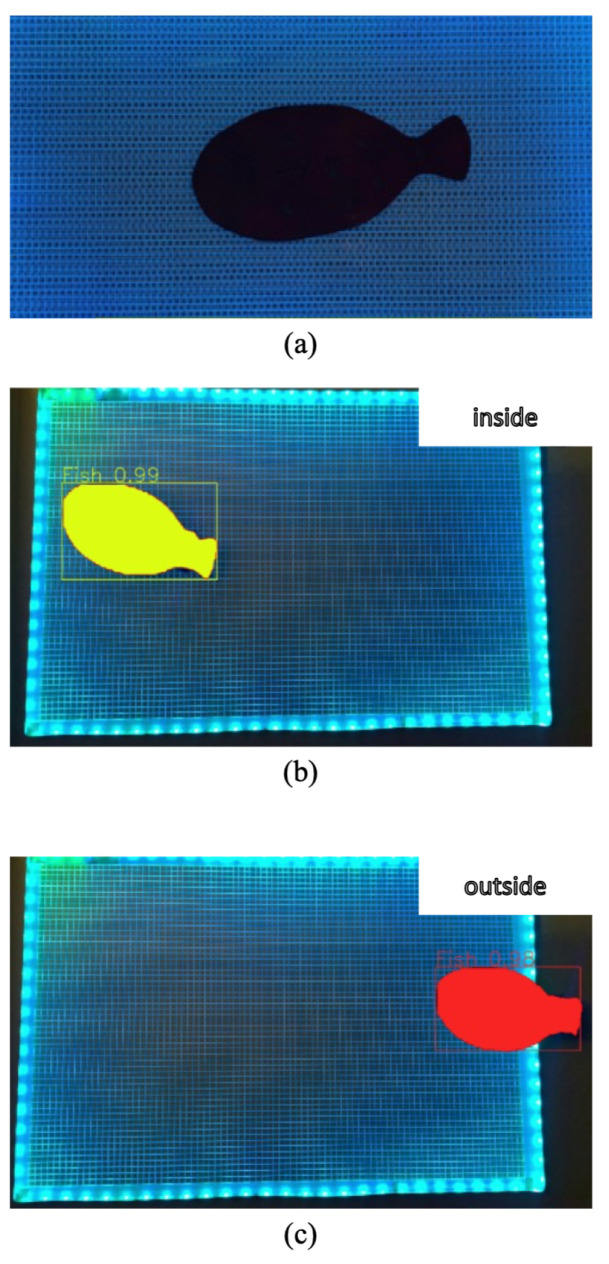
The results of the one raw image (**a**) and two images with the instance segmentation (**b**,**c**) under the low light condition. (**a**) represents the raw image, (**b**,**c**) represent each result inside and outside the grid.

Figure 5 shows the results of the one raw image and two images with the instance segmentation under the low light condition. (a) represents the raw image, (b) and (c) represent each result inside and outside the grid panel. The two different colors indicate the different locations of the mockup fish. The results make clear that it is possible to recognize the location of the mockup fish through the instance segmentation results despite the dark environment.

**Figure 6:**
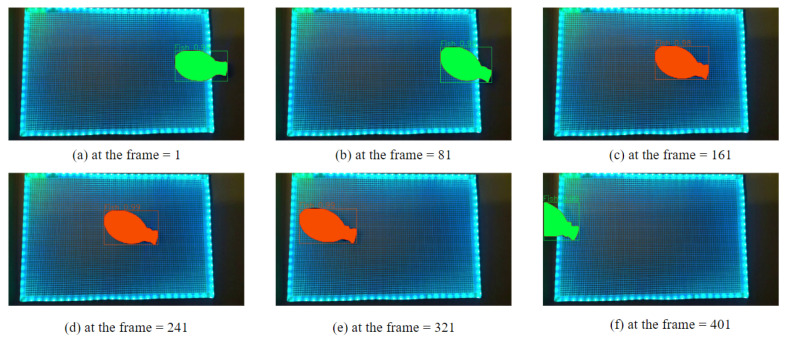
Results of the real-time continuous instance segmentation using the single RGB web camera (from left to right, top to bottom): the green-colored mockup fish represents the location outside the grid, and the orange-colored fish represents the inside of the grid (https://youtu.be/6s0JH-8w4o8, accessed on 14 November 2023).

Figure 6 shows the real-time continuous instance segmentation results using the single RGB web camera (from left to right, top to bottom). The green-colored mockup fish represents the location outside the grid panel, and the orange-colored fish represents inside the grid. The results show the effectiveness of the segmentation model throughout the entire video and accurately predict object labels and coordinates. Furthermore, the results show that our approach can track them in real time (in all the different frames) to predict the necessary information for size and quantity estimation. From that, we get the object’s mask and grid information to apply to the algorithm to estimate the length and calculate the number of fish in the water tank.

### 4.2. Results of Measurement with Instance Segmentation

**Figure 7:**
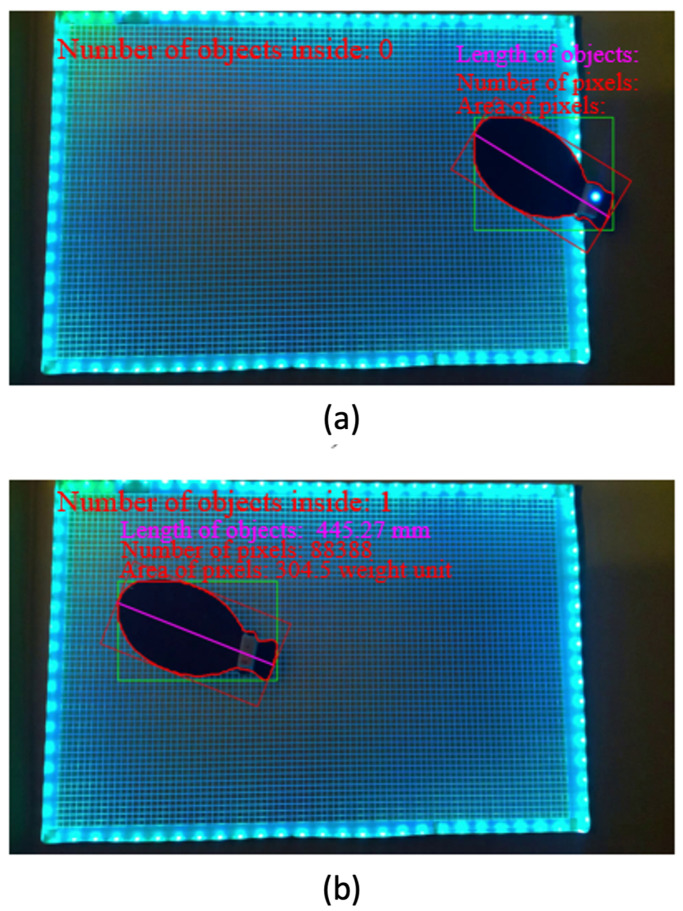
Results of the estimated fish length and the number of fish inside the grid: when the mockup fish exists outside the grid, it is not necessary to estimate the length and count the number of fish (**a**). When the mockup fish is inside the grid, the results show the estimated length while converting the number of pixels to the millimeter unit and the number of target fish (**b**) (https://youtu.be/w2CnC5Q6NcM), accessed on 14 November 2023).

Figure 7 shows the results of the estimated fish length and the number of fish inside the grid. When the mockup fish exists outside the grid, estimating the length and counting the number of fish is optional. However, when the mockup fish is inside the grid, the results show the estimated length while converting the number of pixels to the millimeter unit and the number of target fish. The results found that the fish’s estimated size showed some error of ±5 mm (actually 450 mm and estimated 445 mm). There was approximately 2% error.

As a result, the proposed method enabled us to measure the fish size automatically despite the dark environment. Furthermore, the produced error can be improved while regulating the distance between two intersection points of the grid if necessary.

## 5. Discussion

In this study, we proposed the light-emitting grid panel as the metric to estimate the size of objects from images in real time. In contrast, most of the other research using methods such as data collection should include physical information such as length, width, including fins or not, growing time, and weight [22,23]. Instead, our approach focused on analyzing objects in the single RGB web camera and predicting the size without any other information. This point enabled farm managers to reduce the labor force.

Effective management of aquaculture farms requires precise biomass and fish length estimations during rearing. Fish biomass is derived from the total number of fish counted in a specific volume of water multiplied by the average weight of the fish sampled. The exact information for the biomass can help us to predict daily intake demand to avoid under- or overfeeding. Farmers use fish size to track growth rates and plan accurate feeding to minimize water pollution due to excess feeding. In addition, the regular acquisition of fish biomass information has been identified as an urgent requirement for managers to optimize control of stocking densities and ultimately determine the optimal time for harvesting.

Very little equipment can measure the length of fish in real time through devices like the method developed this time. At the fish farm site, the samples are directly captured and anesthetized, then the length is measured using a ruler, and the weight is measured using a scale. Since all fish in an aquarium cannot be measured this way, the method is carried out under the premise that fish in the same aquarium are similar because they grow up eating the same amount of feed. However, even among fish in the same water tank, some consume much feed whereas others do not; thus, the above premise can be considered incorrect. Therefore, this proposed method is expected to reduce labor in fish farms because it can measure length in real time without capturing fish.

## 6. Conclusions

This study related to a technique for automatically measuring the size of fish on a farm using a light-emitting grid panel. This research can be an important study for establishing facilities for smart aquafarms as part of basic research for the automation of the entire cycle of fish farms. Furthermore, our results increase the productivity of fish farms and reduce labor. The targeting technology in this study is the core and will be applied to real fish farms soon.

The processing pipeline suggested in this research can precisely segment individual fish in images obtained during conventional fisheries surveys utilizing the Deep Vision commercially available equipment. The pipeline’s three primary stages are pre-processing segmentation model and gradient refining. Each stage significantly enhances the system’s performance as a whole.

However, this proposed method still has limitations. The water in the tank must be clear and transparent, and the water flow must be calm. Otherwise, the fish image on the grid cannot be accurately measured from the camera. Thus, future research directions can be considered to improve hardware for measuring fish size without a camera. In addition, although the water in the tank is not transparent and there is a water flow, we can also consider research directions that will improve the software on estimating the length of fish from images measured using a camera.

## Data Availability

Data are contained within the article.
